# Predicting pain among female survivors of recent interpersonal violence: A proof-of-concept machine-learning approach

**DOI:** 10.1371/journal.pone.0255277

**Published:** 2021-07-29

**Authors:** Edward Lannon, Francisco Sanchez-Saez, Brooklynn Bailey, Natalie Hellman, Kerry Kinney, Amber Williams, Subodh Nag, Matthew E. Kutcher, Burel R. Goodin, Uma Rao, Matthew C. Morris

**Affiliations:** 1 Department of Psychiatry and Human Behavior, University of Mississippi Medical Center, Jackson, Mississippi, United States of America; 2 Department of Psychology, University of Tulsa, Tulsa, Oklahoma, United States of America; 3 Department of Anesthesiology, Perioperative and Pain Medicine, Stanford University, Stanford, CA, United States of America; 4 School of Engineering and Technology, Universidad Internacional de La Rioja, Logroño, Spain; 5 Department of Psychology, The Ohio State University, Columbus, Ohio, United States of America; 6 Department of Psychiatry, College of Medicine, University of Illinois at Chicago, Chicago, IL, United States of America; 7 Department of Neuroscience and Pharmacology, Meharry Medical Center, Tennessee, United States of America; 8 Department of Surgery, University of Mississippi Medical Center, Jackson, Mississippi, United States of America; 9 Department of Psychology, University of Alabama at Birmingham, Birmingham, Alabama, United States of America; 10 Department of Psychiatry & Human Behavior, Department of Pediatrics, and Center for the Neurobiology of Learning and Memory, University of California–Irvine, Irvine, California, United States of America; 11 Children’s Hospital of Orange County, Orange, CA, United States of America; Universiteit Antwerpen, BELGIUM

## Abstract

Interpersonal violence (IPV) is highly prevalent in the United States and is a major public health problem. The emergence and/or worsening of chronic pain are known sequelae of IPV; however, not all those who experience IPV develop chronic pain. To mitigate its development, it is critical to identify the factors that are associated with increased risk of pain after IPV. This proof-of-concept study used machine-learning strategies to predict pain severity and interference in 47 young women, ages 18 to 30, who experienced an incident of IPV (i.e., physical and/or sexual assault) within three months of their baseline assessment. Young women are more likely than men to experience IPV and to subsequently develop posttraumatic stress disorder (PTSD) and chronic pain. Women completed a comprehensive assessment of theory-driven cognitive and neurobiological predictors of pain severity and pain-related interference (e.g., pain, coping, disability, psychiatric diagnosis/symptoms, PTSD/trauma, executive function, neuroendocrine, and physiological stress response). Gradient boosting machine models were used to predict symptoms of pain severity and pain-related interference across time (Baseline, 1-,3-,6- follow-up assessments). Models showed excellent predictive performance for pain severity and adequate predictive performance for pain-related interference. This proof-of-concept study suggests that machine-learning approaches are a useful tool for identifying predictors of pain development in survivors of recent IPV. Baseline measures of pain, family life impairment, neuropsychological function, and trauma history were of greatest importance in predicting pain and pain-related interference across a 6-month follow-up period. Present findings support the use of machine-learning techniques in larger studies of post-IPV pain development and highlight theory-driven predictors that could inform the development of targeted early intervention programs. However, these results should be replicated in a larger dataset with lower levels of missing data.

## Introduction

Interpersonal violence (IPV) can take the form of intimate partner violence, domestic violence, violent crime, and can involve intimidation/harassment or physical/sexual assault. Women are at especially high risk for exposure to IPV [1–3]. Indeed, results from the WHO Multi-country Study on Women’s Health and Domestic Violence against Women suggest a lifetime prevalence of physical/sexual partner violence between 15%-17% [4]. IPV is a major public health concern and is associated with severe complications including higher rates of injury and disability, negative medical (e.g., respiratory, cardiovascular, and endocrine dysfunction) [5, 6] and mental health outcomes (e.g., posttraumatic stress disorder [PTSD], depression, anxiety), and increased mortality [6].

IPV is strongly associated with the emergence and/or worsening of chronic pain. For example, IPV is related to worse headaches, back pain, pelvic pain, painful intercourse, fibromyalgia, and abdominal pain, and is more generally associated with disabling pain and increased pain severity [7–12]. Daily pain complaints are common in the early aftermath of IPV and associated with increased risk for chronic pain development [13–16]. Although there is a strong association between IPV exposure and risk for chronic pain, under what circumstances and to what extent IPV exposure leads to chronic pain is highly variable [5, 7, 10]. Hence, identifying predictors of pain development after an incident of IPV is crucial to aid in the early detection and prevention of chronic pain.

The combined shared vulnerability and mutual maintenance model of post-injury disability [17] proposes that preexisting biological and psychosocial vulnerabilities place individuals at increased risk of developing both PTSD and chronic pain. Consistent with this model, a range of biopsychosocial factors (e.g., genetic, neurobiological, cognitive, sociodemographic, and environmental) have been implicated in risk for chronic pain [18–23]. However, researchers have yet to identify the most reliable predictors of pain after IPV. Improved prediction of pain can potentially be achieved by simultaneously examining a diverse array of cognitive, behavioral, biological, and environmental factors. Machine-learning (ML) methods can be used to identify patterns from data that enhance predictive performance [24]; these algorithms can handle large, complex data structures and are better-suited to predict the development of pain than general linear models [25]. Indeed, ML models have been previously used on data from the current study to successfully predict PTSD onset in young women who recently experienced interpersonal violence [26].

Past studies have successfully used ML strategies to predict chronic pain symptoms and development (e.g., post-surgical and rheumatoid arthritis) [27–32]. Lötsch and colleagues [27] used a ML approach to successfully categorize patients into persistent pain versus non-persistent pain groups after breast cancer surgery; the most relevant predictors included age, body mass index, depression, and anxiety. Further, Lötsch and colleagues [28] used ML to identify pre-surgical cold pressor task responses as predictors of post-surgical pain. However, ML has not been used to predict the development of pain in IPV survivors.

This proof-of-concept study builds on the extant literature by using a ML approach to identify theory-driven cognitive, behavioral, and biological factors (e.g., coping, disability, psychiatric diagnosis/symptoms, PTSD/trauma, executive function, neuroendocrine, physiological stress response, and acute pain) in recent IPV survivors that are most relevant to pain prediction over a 6-month follow-up period.

## Materials and methods

All procedures were approved by the Meharry Medical College Research Ethics Board. Participants gave oral and written consent to partake in the study. The presented data is a secondary analysis of a larger study investigating predictors of the development of PTSD after a recent IPV event.

### Participants

Participants were young adult women (n = 47), ages 18 to 30, who had experienced IPV (e.g., physical and/or sexual assault, mugging) within three months before their baseline assessment. Although this IPV incident was the focus of PTSD assessments throughout the study, prior trauma exposure was common (mean number of prior traumatic events = 7.0, *SD* = 3.2). Recruitment occurred through online advertisements and research participant registries, local agencies coordinating services for survivors of domestic violence and sexual assault, and through a team of nurse practitioners providing medical legal exams to rape survivors in a local hospital. Exclusion criteria included: active substance use disorder; active major depressive disorder (MDD) that preceded their index trauma; active PTSD resulting from a traumatic event that occurred prior to the three-month window; active/in remission bipolar or psychotic disorder; serious health conditions known to influence hypothalamic-pituitary-adrenal (HPA) activity (e.g., Cushing’s or Addison’s Disease, hyperthyroidism); pregnancy; and current use of prescription (e.g., corticosteroids, antidepressant medication) or non-prescription drugs known to affect HPA activity. Participants were not excluded if they were currently using oral contraceptives. MDD was assessed using the Structured Clinical Interview for DSM-IV [SCID-I]) [33] and PTSD was determined with the CAPS-IV (Clinician Administered PTSD Scale for DSM-IV) [34]. Assessment measures were based on DSM-IV because data collection was initiated prior to the introduction of DSM-5 criteria and creation of validated DSM-5 assessment materials.

### Assessment

Assessments were conducted at baseline, and at 1-, 3-, and 6-month follow-up intervals. Each assessment was conducted over two visits. Semi-structured clinical interviews (i.e., SCID-I, CAPS-IV) were administered at the first visit. Between visits, participants were asked to complete two days of at-home saliva collection to determine diurnal cortisol and alpha-amylase secretion and to complete online questionnaires via Research Electronic Data Capture (REDCap) [35], including the Short-Form McGill Pain Questionnaire (SF-MPQ) [36] and PROMIS (Patient-Reported Outcomes Measurement Information System) Pain Interference Short Form [37]. On the second visit, a modified version of the Trier Social Stress Test (TSST) [38] was administered. The present study reports data on baseline predictors and on pain outcomes assessed at baseline and over the follow-up periods.

### Predictors & outcomes

Predictors from nine categories were assessed: sociodemographic, coping, disability, psychiatric diagnosis/symptoms, PTSD/trauma, executive function, neuroendocrine, physiological stress response, and pain. All outcomes and the majority of predictors were assessed at all time points (Baseline, 1-, 3-, 6- Follow up). For our current analysis, all 4 time points of the SF-MPQ overall and pain interference were used while only the baseline data of all other variables were used.

#### Sociodemographic

Age, race, ethnicity, height, weight, marital status, household income, and years of education were collected through self-report.

#### Coping

Primary/secondary control coping, disengagement coping, and involuntary engagement/disengagement were assessed with the Response to Stress Questionnaire (RSQ) [39]. The RSQ is a 57-item measure where each item is scored on a scale of 1 (Not at all) to 4 (A lot). The RSQ has good internal consistency (α = .73 to .85) and construct validity [39].

Pain catastrophizing was determined by the Pain Catastrophizing Scale (PCS) [40]. The PCS is a 13-item scale that has a total score and 3 subscale scores (rumination, magnification, helplessness). The PCS has adequate internal consistency (total α = .87, rumination α = .87; magnification α = .66, and helplessness α = .78).

#### Disability

Impairment experienced at work/school and/or in the social and family spheres due to trauma-related symptoms was assessed with the Sheehan Disability Scale (SDS) [41]. Each subscale of the SDS ranges from 0 (not at all) to 10 (extremely) with higher scores indicating increased disruption. The SDS has adequate sensitivity (.83) and specificity (.69) [42]. Health service utilization (i.e., medical and mental health treatment) in the previous three months was assessed via self-report.

#### Psychiatric diagnosis/symptoms

The SCID-I [33] assessed current and past diagnoses, age of onset, and number of episodes for mood and anxiety disorders. Anxiety was assessed using the Generalized Anxiety Disorder 7-item scale (GAD-7) [43] (scores ranging from 0 to 21; α = .83 - .93) [44] and the State-Trait Anxiety Inventory (only the trait version was used) [45] (Reliability = .65 - .86) [45]. Current depression severity was assessed with the Beck Depression Inventory second edition (BDI-II) [46]. The BDI-II is a validated, highly reliable (α = .93 among college students, α = .92 among outpatients) 21-item scale with scores ranging from 0 to 63 [46, 47].

#### PTSD/trauma

Posttraumatic stress symptom severity was assessed with the CAPS-IV interview [34] and the self-report PTSD Checklist for DSM-IV [48]. Number of prior traumatic life events was assessed using the Life Events Checklist (LEC) [49]. The LEC is a reliable (mean item Kappa = .61; Test-Retest r = .82) and valid measure [49]. Dissociation during trauma was assessed with the Peri-traumatic Dissociative Experiences Questionnaire (PDEQ) [50]. The PDEQ is a 10-item, 5-point-Likert-scaled, and is the most widely used self-report measure of peri-traumatic dissociation. High scores on the PDEQ are strongly associated with PTSD and MDD symptoms, general dissociative tendencies, and severity of trauma [51].

Childhood abuse and neglect were assessed with the Childhood Trauma Questionnaire (CTQ) [52]. The CTQ has five subscales: emotional abuse, physical abuse, sexual abuse, emotional neglect, and physical neglect. The CTQ has good reliability (intraclass correlation = .88) and validity (Factors α = .79 - .94) [52]. Stress levels for major life events and daily hassles were assessed with the young adult social stress version of the Perceived Events Scale (PES) [53]. Participants were asked to indicate whether each event occurred during this time, and to rate the valence of those events on a 9-point scale (-4 = Extremely Bad; +4 = Extremely Good). A total score for negative events occurring in the past 6 months was calculated by summing across all events rated -1 to -4 on desirability. Total recent stress level scores were multiplied by -1, so that higher scores indicate higher stress levels.

Trauma-related cognitive appraisals (self, world, and self-blame) were assessed with the Posttraumatic Cognitions Inventory (PTCI) [54]. The PTCI Is a 33-item instrument with good validity and reliability (α = .97; Test-Retest reliability = .74) [54].

#### Executive function

Abstract reasoning, set shifting, and problem solving were assessed using the Wisconsin Card Sorting Test (WCST) [55]. Set shifting abilities (Trails), cognitive flexibility (Design Fluency), and inhibition (Color-Word) were determined using selected Delis–Kaplan Executive Function System (D-KEFS) subtests [56].

#### Neuroendocrine secretion

Diurnal cortisol and alpha-amylase were determined with saliva samples collected at home (Sarstedt Inc., Netwon, NC) at five established times (waking, 30 minutes after awakening, before lunch, 3pm, and 9pm or bedtime) over two consecutive days (See assay details in Physiological Stress Response section). Daily output was computed using the area under the curve with respect to ground (AUCg) formula [57]; average AUCg was determined across both collection days. Cortisol and alpha-amylase awakening responses were determined by the mean difference, across both collection days, from awakening to 30 minutes after awakening. Diurnal cortisol and alpha-amylase slopes were averaged individualized regression coefficients for each participant’s daily samples [58]. Prolonged HPA secretion was determined by hair cortisol concentrations from 3cm hair segments as described previously [59]. Intra- and inter-assay coefficients of variation for this assay are below 12%.

#### Physiological stress response

The Trier Social Stress Test (TSST) [38], a social-evaluative stress task, was used to induce a stress response. Cortisol and alpha-amylase reactivity were determined using four pre-TSST [38] samples collected at 30-minute intervals and seven post-TSST samples collected at 10-minute intervals. Reactivity index was the difference between cortisol/alpha-amylase on the final pre-TSST sample and the maximum post-TSST level. For diurnal secretion and reactivity measures, free cortisol levels were determined by commercial chemiluminescence immunoassay [60] and alpha-amylase levels were determined by a quantitative enzyme kinetic method [61]. Intra- and inter-assay coefficients of variation for these assays were below 6%. Throughout the TSST, we continuously recorded heart rate using a Polar H7 chest-strap (Polar, Finland) and Actigraph wrist watch; we computed mean resting heart rate (over a TSST 20-minute period prior to the instructions), anticipatory heart rate (over 5 minutes immediately preceding the TSST), mean heart rate during the 10-minute TSST, and recovery heart rate (over a 20-minute period immediately following the TSST).

#### Pain

Self-reported sensory/affective pain and current pain intensity (Visual Analog Scale [VAS]) were determined by the Short-Form McGill Pain Questionnaire (SF-MPQ) [36]. The SF-MPQ has adequate test-retest reliability (.62-.95) [62]. Pain severity was measured by the SF-MPQ Overall subscale of the SF-MPQ. Pain-related functional disability over the week prior to assessment was assessed with the PROMIS Pain Interference Short Form [37]. The PROMIS pain interference uses item response theory and has excellent psychometric properties in its item bank including reliability equivalent to .96-.99 in a subset of scores [63].

### Data analysis

#### Data preprocessing

All variables were examined for distributional properties and cases were screened for univariate outliers. Three outliers were detected for diurnal cortisol and alpha-amylase levels, which were Winsorized at three standard deviations [64]. Missing values in predictor data were imputed using Classification and Regression Trees (CART) proximity algorithm method [65] of the Multivariate Imputation by Chained Equations (MICE) library [66] in R [67]. To control for the variability in assessment timing between participants, two variables were constructed: 1) Days since trauma and 2) Days since baseline evaluation.

#### Ensemble machine-learning

Two Gradient Boosting Machine (GBM) models were used to predict pain interference, and SF-MPQ overall pain scores over follow-up from baseline data. GBM models assemble small decision trees to build prediction models for regression and classification problems. Gradient boosting adds models sequentially and trains weak models with corrections learned previously. Gradient boosting in combination with cross-validation provides slow learning accurate models with protections against overfitting. Baseline and SF-MPQ overall pain and pain interference scores were included in all models. SF-MPQ overall was treated as a binomial classification model (0: SF-MPQ overall pain = no pain or mild pain, n = 65; 1: SF-MPQ overall pain = discomforting pain, distressing pain, horrible pain or excruciating pain, n = 39) whereas pain interference was conducted as a regression model. Two nested repeated cross-validation models were constructed (Inner loops had 3 repetitions with 4-folds; Outer loops had 3 repetitions with 5-folds). GBM models were conducted using library “xgboost” [68] in R [67]. The hyperparameters of the GBM models (depth of trees, minimum child weight, and number of rounds) [24] were chosen using the inner loop mean squared error estimations. The minimum child weight is the minimum sum of instance weight needed in a child. If the tree partition step results in a leaf node with the sum of instance weight less than minimum child weight, then the building process will stop further partitioning. When instances are equally weighted, this simply corresponds to minimum number of instances needed to be in each node. The larger the minimum child weight, the more conservative the algorithm will be [24].

## Results

### Sample characteristics

**Fig 1:**
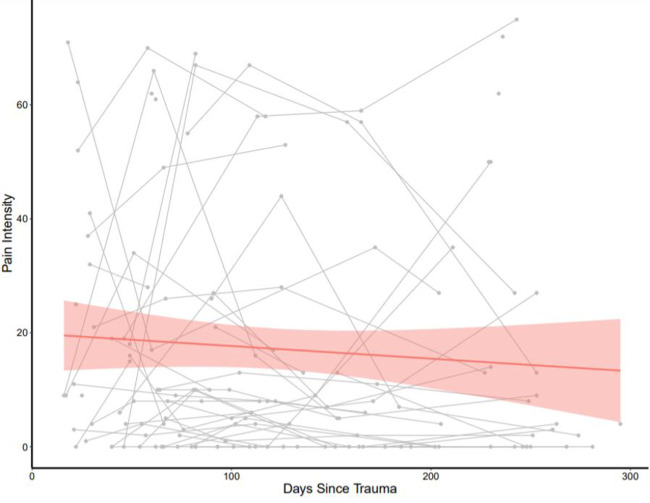
Reduction of pain intensity Short-Form McGill Questionnaire visual analog scale over time in young women with recent interpersonal violence. Red shade represents 95% CI. Gray lines represent individual changes in pain interference over time.

**Fig 2:**
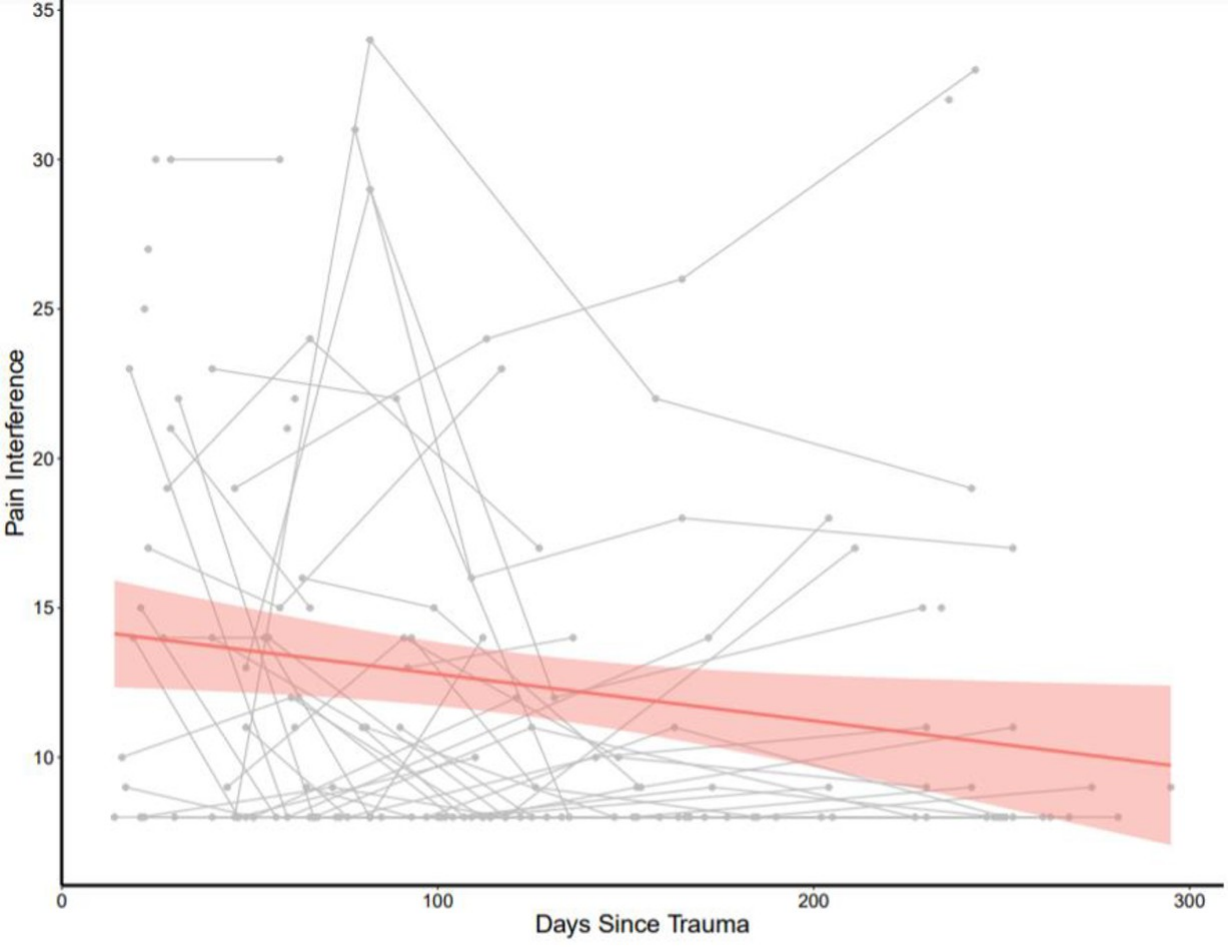
Reduction of pain interference over time in young women with recent interpersonal violence. Red shade represents 95% CI. Gray lines represent individual changes in pain interference over time.

The mean age of participants was 24.2 years (*SD* = 3.4; range = 18 to 30). Participants included 24 White/Caucasian (51%), 13 Black/African American (27%), 6 Asian (12%), and 4 Hispanic (8%) women. Most women were single (83%) and reported a mean of 15 years of education (*SD* = 4) (See Table 1 for a summary of missing demographic/ clinical information and data). The mean duration from index trauma to baseline assessment was 45.1 days (*SD* = 24.6 days). Overall, and SF-MPQ VAS Pain Intensity (See Fig 1) and Pain interference reduce over time (See Fig 2).

**Table 1:** Descriptive and clinical characteristics and missing data of recent interpersonal violence survivors.

	Mean (SD) or n (%)	Missing n (%)
Sociodemographic		
Age (years)	24.2 (3.36)	0 (0%)
Race/ethnicity		0 (0%)
Asian	6 (12.77%)	
Black/African American	13 (27.66%)	
White/Caucasian	24 (51.06%)	
Hispanic	4 (8.51%)	
Education (years)	14.7 (1.91)	4 (8.5%)
Marital Status		1 (2.1%)
Single	39 (82.98%)	
Married	1 (2.13%)	
Engaged	2 (4.26%)	
Living with partner	4 (8.51%)	
Disability		
Impairment family life (SDS)	2.00 (2.65)	0 (0%)
Impairment social life (SDS)	2.21 (2.32)	0 (0%)
Impairment school/work (SDS)	2.32 (2.49)	0 (0%)
Coping		
Primary control (RSQ)	.197 (.0429)	1 (2.1%)
Secondary control (RSQ)	.255 (.0466)	1 (2.1%)
Disengagement (RSQ)	.143 (.0263)	1 (2.1%)
Pain catastrophizing	11.7 (9.35)	0 (0%)
Psychiatric Diagnoses/Symptoms		
Major depression (current)	5 (10.64%)	0 (0%)
Number of prior MDEs	3.15 (4.04)	0 (0%)
GAD (current)	8 (17.02%)	0 (0%)
Panic Disorder (current)	2 (4.26%)	0 (0%)
Agoraphobia (current)	1 (2.13%)	0 (0%)
Depressive severity (current; BDI-II)	15.3 (10.9)	1 (2.1%)
GAD severity (current; GAD-7)	6.78 (5.43)	1 (2.1%)
Trait anxiety (STAI)	45.8 (10.2)	1 (2.1%)
PTSD/Trauma		
PTCI–self	2.81 (1.11)	1 (2.1%)
PTCI–world	4.32 (1.14)	1 (2.1%)
PTCI–self-blame	3.39 (1.07)	1 (2.1%)
Peri-traumatic dissociation (PDEQ)	21.9 (7.61)	1 (2.1%)
Stress levels major events (PES)	6.41 (3.22)	1 (2.1%)
Stress levels daily hassles (PES)	36.8 (9.31)	1 (2.1%)
PTSD diagnosis (current)	37 (78.72%)	0 (0%)
PTSD severity baseline (CAPS-IV total)	47.3 (23.9)	0 (0%)
PTSD Checklist Total (PCL)	41.4 (13.7)	1 (2.1%)
Childhood trauma Total (CTQ)	47.0 (15.6)	1 (2.1%)
Emotional abuse (CTQ)	11.5 (5.29)	1 (2.1%)
Physical abuse (CTQ)	8.6 (3.95)	1 (2.1%)
Sexual abuse (CTQ)	7.7 (4.21)	1 (2.1%)
Emotional neglect (CTQ)	10.5 (4.63)	1 (2.1%)
Physical neglect (CTQ)	8.8 (2.75)	1 (2.1%)
Number of traumatic events (LEC)	7.0 (3.21)	0 (0%)
Cumber of traumatic categories (LEC)	3.4 (1.56)	0 (0%)
Days since trauma	48.5 (25.5)	0 (0%)
Days since baseline evaluation	98.6 (60)	0 (0%)
IPV type		0 (0%)
Physical assault	22 (46.81%)	
Sexual assault	14 (29.79%)	
Physical + sexual assault	2 (4.26%)	
Inappropriate touch/harassment	3 (6.38%)	
Weapon threat/mugging/captive	6 (12.77%)	
Executive Function		
Total Correct (WCST)	67.0 (10.76)	4 (8.5%)
Learning-to-learn index (WCST)	-3.06 (8.34)	6 (12.8%)
Conceptual responses (WCST)	62.6 (14.30)	4 (8.5%)
TM switching vs. number (D-KEFS)	9.13 (1.83)	1 (2.1%)
Design Fluency Filled Dots (D-KEFS)	11.2 (2.7)	1 (2.1%)
CW inhibition/switching (D-KEFS)	10.3 (3.70)	3 (6.4%)
Neuroendocrine Secretion		
Cortisol AUCg	4140 (2580)	2 (4.3%)
Cortisol awakening response	1.56 (6.13)	0 (0%)
Cortisol diurnal slope	-.629 (.294)	0 (0%)
Alpha-amylase AUCg	60400 (39100)	5 (10.6%)
Alpha-amylase awakening response	.993 (24.3)	1 (2.1%)
Alpha-amylase diurnal slope	.376 (.416)	
Hair cortisol concentration	16.7 (13.5)	28 (59.6%)
Physiological Stress Response		
TSST		
Pre-stress cortisol	3.62 (10.4)	4 (8.5%)
Cortisol reactivity	2.31 (4.77)	6 (12.8%)
Pre-stress alpha-amylase	87.3 (68.6)	6 (12.8%)
Alpha-amylase reactivity	57.7 (79.2)	7 (14.9%)
Recovery Heart Rate	14.5 (9.36)	10 (27%)
Pain		
Baseline		
Pain interference (PROMIS)	14.3 (6.94)	0 (0%)
SF-MPQ Sensory	4.9 (5.42)	0 (0%)
SF-MPQ Affective	2.1 (2.07)	0 (0%)
SF-MPQ VAS	16.7 (20.74)	3 (6.4%)
SF-MPQ Overall		0 (0%)
No/Mild Pain	46 (97.87%)	
Discomforting/Distressing/Horrible/Excruciating	1 (2.13%)	
1-Month		
Pain interference (PROMIS)	12.5 (7.05)	8 (17.0%)
SF-MPQ Sensory	3.11 (3.98)	8 (17.0%)
SF-MPQ Affective	.80 (0.96)	8 (17.0%)
SF-MPQ VAS	14.55 (19.8)	10 (4.7%)
SF-MPQ Overall		8 (17.0%)
No/Mild Pain	32 (68.08%)	
Discomforting/Distressing/Horrible/Excruciating	7 (14.9%)	
3-Month		
Pain interference (PROMIS)	10.7 (4.76)	12 (25.5%)
SF-MPQ Sensory	3.11 (3.9)	12 (25.5%)
SF-MPQ Affective	.80 (0.96)	12 (25.5%)
SF-MPQ VAS	14.55 (19.8)	14 (48.9%)
SF-MPQ Overall		12 (25.5%)
No/Mild Pain	32 (68.08%)	
Discomforting/Distressing/Horrible/Excruciating	7 (14.9%)	
6-Month		
Pain interference (PROMIS)	11.8 (6.64)	17 (36.2%)
SF-MPQ Sensory	3.1 (4.69)	17 (36.2%)
SF-MPQ Affective	1.3 (2.39)	17 (36.2%)
SF-MPQ VAS	17.1 (22.84)	17 (36.2%)
SF-MPQ Overall		17 (36.2%)
No/Mild Pain	25 (53.19%)	
Discomforting/Distressing/Horrible/Excruciating	5 (10.64%)	

PTSD = posttraumatic stress disorder; PTCI = Posttraumatic Cognitions Inventory; RSQ = Responses to Stress Questionnaire; WCST = Wisconsin Card Sorting Test; D-KEFS = Delis-Kaplan Executive Function System; AUC_g_ = area under the curve with respect to ground; SF-MPQ = Short-Form McGill Questionnaire; PROMIS = Patient-Reported Outcomes Measurement Information System; GAD = generalized anxiety disorder; GAD-7 = Generalized Anxiety Disorder 7-item scale; PES = Perceived Events Scale; MDE = Major depressive episode; BDI-II = Beck Depression Inventory second edition; STAI = State-Trait Anxiety Inventory; PDEQ = Peri-traumatic Dissociative Experiences Questionnaire; CTQ = Childhood trauma Questionnaire; LEC = Life Events Checklist; SDS = Sheehan Disability Scale; CAPS-IV = Clinician Administered PTSD Scale for DSM-IV.

### Predictors of pain scores

From the GBM models the most relevant predictors of pain interference and MPQ overall pain were obtained.

The pain interference model characteristics (*Mean Max Depth* = 6.27, *SD* = 2.40; *Mean Child Weight* = 6.73, *SD* = 1.39; *Mean number of rounds* = 174.27, *SD* = 13.82) suggest adequate performance (Train *RMSE* = 2.29, *R*^2^ = .86; Test *RMSE* = 4.83, *R*^2^ = .31). The five most important predictors included: baseline pain interference (PROMIS), family life impairment (SDS), childhood emotional abuse (CTQ), pain intensity (SF-MPQ VAS), sensory pain (SF-MPQ) (See Table 2).

**Table 2:** Relevant features (>1% importance) in gradient boosting machine models predicting pain interference.

Feature by Category		Importance (%)	∑ Importance (%)
Trauma	Childhood Emotional Abuse (CTQ)	6.06	17.37
	Trauma Categories (LEC)	2.62	
	Days since baseline	2.51	
	Childhood Physical Abuse (CTQ)	2.22	
	Dissociation (PDEQ)	1.62	
	PTSD Severity (PCL)	1.19	
	Days since trauma	1.15	
Pain	Pain Interference (PROMIS)	7.22	15.64
	Pain intensity (SF-MPQ VAS)	5.37	
	Sensory Pain (SF-MPQ)	3.05	
Disability	Family Life Impairment (SDS)	6.98	9.34
	Social Life (SDS)	1.33	
	Work/School (SDS)	1.03	
Neuroendocrine Response	Cortisol AUCg	2.91	7.06
	Alpha Amylase Diurnal Slope	1.54	
	Alpha Amylase AUCg	1.34	
	Cortisol Awakening Response	1.27	
Psychiatric Diagnosis/Symptoms	GAD Total (GAD-7)	2.52	2.52
Coping	Secondary Coping (RSQ)	2.37	2.37
Physiological Stress Response	Recovery Heart Rate (TSST)	1.93	1.93
Demographic	Weight	1.21	1.21
Executive Function	Conceptual Responses (WCST)	1.15	1.15

PROMIS = Patient-Reported Outcomes Measurement Information System; SF-MPQ = Short-Form McGill Questionnaire; VAS = Visual Analog Scale; CTQ = Childhood trauma Questionnaire; LEC = Life Events Checklist; PDEQ = Peri-traumatic Dissociative Experiences Questionnaire; PCL = PTSD Checklist for DSM-IV; SDS = Sheehan Disability Scale; AUCg = area under the curve with respect to ground;; GAD = generalized anxiety disorder; GAD-7 = Generalized Anxiety Disorder 7-item scale; RSQ = Responses to Stress Questionnaire; TSST = Trier Social Stress Test; GAD = generalized anxiety disorder; WCST = Wisconsin Card Sorting Test.

The SF-MPQ overall model characteristics (*Mean Max Depth* = 6.47, *SD* = 1.92; *Mean Minimum Child Weight* = 2.13, *SD* = .99; *Mean number of rounds* = 175.67, *SD* = 11.82) suggests excellent performance (Train *AUC* = .98, Accuracy = .92; Test *AUC* = .81, Accuracy = .76). The five most important predictors included: baseline pain interference (PROMIS), pain intensity (SF-MPQ VAS), days since trauma, conceptual responses (WCST), sensory pain (SF-MPQ) (See Table 3 for results & S1 Table for confusion matrix).

**Table 3:** Relevant features (>1% importance) in gradient boosting machine models predicting SF-MPQ overall.

Feature by Category		Importance (%)	∑ Importance (%)
Pain	Pain interference (PROMIS)	10.17	25.35
	Pain intensity (SF-MPQ VAS)	10.13	
	Sensory Pain (SF-MPQ)	3.42	
	Affective Pain (SF-MPQ)	1.63	
Trauma	Days Since Trauma	5.05	14.89
	Days Since Baseline	1.81	
	Self-Blame (PTCI)	1.67	
	Childhood Physical Neglect (CTQ)	1.66	
	Childhood Sexual Abuse (CTQ)	1.41	
	CTQ Total (CTQ)	1.02	
	IPV type—Sexual Assault	1.15	
	Broad Trauma	1.12	
Executive Function	Conceptual Responses (WCST)	4.84	8.54
	Filled Dots SS (DF)	1.91	
	Total Correct (WCST)	1.79	
Neuroendocrine Response	Hair Cortisol Concentrations	3.23	6.94
	Cortisol AUCg	2.51	
	Alpha Amylase AUCg	1.2	
Physiological Stress Response	Pre-TSST Cortisol (TSST)	1.25	2.34
	TSST Cortisol Reactivity (TSST)	1.09	
Psychiatric Diagnosis/Symptoms	GAD Total (GAD-7)	1.73	1.73
Demographic	Height	1.62	1.62
Disability	Family Life Impairment (SDS)	1.29	1.29

PROMIS = Patient-Reported Outcomes Measurement Information System; SF-MPQ = Short-Form McGill Questionnaire; VAS = Visual Analog Scale; WCST = Wisconsin Card Sorting Test; DF = Design Fluency of the Delis–Kaplan Executive Function System (D-KEFS); PTCI = Posttraumatic Cognitions Inventory; CTQ = Childhood trauma Questionnaire; CAPS-IV = Clinician Administered PTSD Scale for DSM-IV; AUCg = area under the curve with respect to ground; TSST = Trier Social Stress Test; GAD = generalized anxiety disorder; GAD-7 = Generalized Anxiety Disorder 7-item scale; SDS = Sheehan Disability Scale.

## Discussion

Chronic pain is a major global health problem [69]. Interpersonal violence (IPV) is associated with the emergence and/or worsening of pain [7–12]. However, there is a critical gap in our understanding of the factors that predict worsening pain in IPV survivors. This proof-of-concept longitudinal study of recent IPV survivors used a machine-learning (ML) approach to investigate baseline cognitive and neurobiological predictors of daily pain and pain-related interference across 1-, 3-, and 6-month follow-up assessments. Consistent with the combined shared vulnerability and mutual maintenance model of posttraumatic pain [17], results identified important biological and psychosocial predictors of pain severity and pain-related interference. This study demonstrates the promise of ML approaches to pain prediction among IPV survivors, highlights the potential relevance of risk and protective actors across multiple domains, and indicates the need for larger samples to replicate findings in order to enhance pain prediction in IPV survivors.

Prediction models for pain severity and pain-related interference performed well when compared to prior ML studies of pain. One study predicting follow-up markers of disease severity (e.g., number of swollen joints) in people with rheumatoid arthritis achieved an AUC of .658 [30]. Another study found that a six item tool was able to classify postoperative pain after breast surgery with a sensitivity of 33% and specificity of 95% [70]. An ML approach was subsequently used to predict postsurgical pain among breast surgery patients, which resulted in models with 79% sensitivity and 51% specificity for correct assignment and 95% negative predictive power [27]. Together, these studies support the promise of ML approaches as tools for pain prediction.

The present findings suggest two key variable domains that are important in predicting the development of pain among recent IPV survivors: pain characteristics and trauma history. Pain-related variables were strong predictors in both the SF-MPQ overall pain and pain interference models. This is in line with research showing that baseline (acute) pain is a strong predictor of future (chronic) pain. For example, a large cohort study (n = 386) of patients with a distal radius fracture found that baseline pain was a strong predictor of future pain and function at a 1-year follow-up [71]. A study of individuals with rheumatoid arthritis (n = 95) found that current pain was the strongest predictor of pain at 1-year follow-up [72]. Another study of patients with recent lower back pain episodes (n = 174) found that baseline pain intensity was the most important predictor of future pain [73]. Finally, a study investigating the chronification of lower back pain noted that the strongest predictor of pain disability at follow-up was baseline pain disability [21].

Trauma-related variables emerged as important predictors of SF-MPQ overall pain and pain interference. Past research suggests a strong link between PTSD and pain [17]. IPV worsens headaches, back pain, pelvic pain, painful intercourse, fibromyalgia, and abdominal pain [7–12]. Among the strongest trauma-related predictors in our models were childhood emotional abuse and days since trauma. There is strong evidence for a link between pain and childhood abuse/neglect [74]. One meta analytic study found that chronic pain patients were more likely to report childhood abuse/neglect (N = 1,811), and chronic pain patients who reported childhood abuse/neglect endorsed worse pain-related symptoms than those who did not (N = 3,040) [74]. Days since the index IPV trauma was an important predictor of pain intensity. This is in line with the literature suggesting pain is common following a traumatic event but that resilience/recovery is the normative response [75]. The World Health Organization World Mental Health Surveys (26 surveys in 24 countries) indicated that ~70% of the world adult population has experienced a traumatic event, yet only 5.6% of those exposed have PTSD [76]. Similar to research on PTSD prediction, identifying recent IPV survivors who will develop pain and could benefit from preventive interventions remains a significant challenge.

ML models identified executive function (EF) and disability as important variable domains to consider for pain prediction in recent survivors of IPV. Models indicate that EF, assessed via the Wisconsin Card Sorting Task (WCST; Total and Conceptual Responses) [55] and D-KEFS Design Fluency (Filled Dot), strongly predicted pain severity. There are several possible pathways through which EF could influence pain. EF may influence the selection and implementation of pain-related coping skills. The importance of EF to coping and pain outcomes in youth with chronic pain conditions has been previously established [77–79]. Stronger EF may also allow for greater sustained attention, which has been shown to lower pain ratings in dual-task paradigms where participants perform executive functioning tasks while in pain [80–82]. Disability was also a strong predictor of pain interference. In particular, the second strongest predictor of pain interference (6.98% importance) was the family impairment subscale of the Sheehan Disability Scale [41]. The family impairment subscale assesses the degree to which trauma exposure has disrupted family/home responsibilities, while the overall test also includes additional measures of functional impairment across work/school and social domains.

The present study has several strengths, including its focus on young adult women at elevated risk for IPV and the comprehensive assessment of theory-driven predictors. To our knowledge this study is the first to apply ML to identify theory-driven predictors of pain outcomes in survivors of a recent IPV. Assessment of cognitive predictors included volitional coping efforts [39] in addition to measures of EF and cognitive flexibility [55, 56]. An extensive battery of physiological/neuroendocrine stress response markers was used to capture diurnal cortisol secretion and TSST reactivity [38], diurnal alpha-amylase secretion and TSST reactivity, as well as prolonged HPA activation assessed via hair cortisol concentrations. PTSD diagnostic status was assessed using a well-validated semi-structured interview (CAPS-IV) [34]. Results suggest that using ML approaches in this population to detect the emergence of pain is a viable strategy for future research.

The present study used state-of-the-art ML methods to model potential pain-relevant predictors across multiple biological and psychosocial domains. This approach would not be feasible with general linear models. Although prior studies have utilized ML approaches with similar sample sizes [83], the present findings should be considered a proof-of-concept until replicated in larger samples due to the high ratio of features to participants. This sample mainly endorsed experiencing an IPV including physical violence (~80%). Future studies are needed to evaluate relations between nonphysical forms of IPV (e.g., emotional or psychological abuse) and pain outcomes. This study did not assess chronic pain status at baseline or across follow-up; we cannot rule out the potential confounding effects of pre-IPV pain. The sample was composed of young adult women exposed to IPV; results may not generalize to populations with different demographic characteristics or types of trauma exposure. Although recommended approaches were implemented for missing data imputation[65], results should be interpreted with caution due to higher levels missing data for a subset of variables (e.g., hair cortisol concentrations). Indeed, future studies may benefit from using attrition mitigating tasks. For example, Fumagalli and colleagues conducted a large RCT focused on attrition reduction strategies and concluded that sending change-of-address forms between assessment times increased retention while providing tailored assessments by target demographic (e.g., age) increased retention [84].

In summary, the present proof-of-concept-study used a longitudinal ML approach to identify biological and psychosocial predictors of pain severity and pain-related interference in women with recent IPV exposure. Results suggest that initial pain, prior trauma exposure, EF, and disability, were among the most important predictors of pain across the 6-month follow-up period. Due to the levels of missing data and sample size of the current study, results should be replicated since it is essential to enhance prediction of chronic pain following IPV in order to mitigate its enormous toll on survivors and society.

## Supporting information

S1 Table Confusion matrix for repeated cross-validation of SF-MPQ overall. Cross validation outer loop consists of 3 repetitions of 5 folder cross validation. Train confusion matrix size is 12 · original set size = 12 · 104 = 1248, Train Accuracy = 751+396/ 1248 = .92. Train MMCE (1-Accuracy) = .08. Test confusion matrix size is 3 original set size = 3 104 = 312. Test Accuracy = 163+73/ 312 = .76. Test MMCE (1- Accuracy) = .24.
(DOCX)

S1 Data (SAV)
