# Supplementary material for: Predicting pain among female survivors of recent interpersonal violence: A proof-of-concept machine-learning approach
Source: PLoS One. 2021 Jul 29;16(7):e0255277. doi: 10.1371/journal.pone.0255277 (PMC8320990; doi:10.1371/journal.pone.0255277)
Supplement: S1 Table — Cross validation outer loop consists of 3 repetitions of 5 folder cross validation. Train confusion matrix size is 12 · original set size = 12 · 104 = 1248, Train Accuracy = 751+396/ 1248 = .92. Train MMCE (1-Accuracy) = .08. Test confusion matrix size is 3 original set size = 3 104 = 312. Test Accuracy = 163+73/ 312 = .76. Test MMCE (1- Accuracy) = .24. (DOCX) [file pone.0255277.s001.docx]

**S1 Table. Confusion Matrix for Repeated Cross-validation of MPQ Overall.**

| **Train** | **Predicted** | |  | **Test** | **Predicted** | |
| --- | --- | --- | --- | --- | --- | --- |
| **True** | **Neg (0)** | **Pos (1)** |  | **True** | **Neg (0)** | **Pos (1)** |
| **Neg (0)** | 751 | 29 |  | **Neg (0)** | 163 | 32 |
| **Pos (1)** | 72 | 396 |  | **Pos (1)** | 44 | 73 |

Cross validation outer loop consists of 3 repetitions of 5 folder cross validation. Train confusion matrix size is 12 · original set size = 12 · 104 = 1248, Train Accuracy = 751+396/ 1248 = 0.92. Train MMCE (1-Accuracy) = 0.08. Test confusion matrix size is 3 · original set size = 3 · 104 = 312. Test Accuracy = 163+73/ 312 = 0.76. Test MMCE (1- Accuracy) = 0.24.
